# Editorial: Traumatic Brain Injury: From Bench to Bedside

**DOI:** 10.3389/fneur.2019.01214

**Published:** 2019-11-15

**Authors:** Aline Silva Miranda, Leonardo Cruz de Souza, Antônio Lúcio Teixeira

**Affiliations:** ^1^Laboratório de Neurobiologia, Departamento de Morfologia, Instituto de Ciências Biológicas, UFMG, Belo Horizonte, Brazil; ^2^Laboratório Interdisciplinar de Investigação Médica, Faculdade de Medicina, Universidade Federal de Minas Gerais, Belo Horizonte, Brazil; ^3^Neuropsychiatry Program, Department of Psychiatry and Behavioral Sciences, McGovern Medical School, University of Texas Health Science Center at Houston, Houston, TX, United States; ^4^Santa Casa BH Ensino e Pesquisa, Belo Horizonte, Brazil

**Keywords:** traumatic brain injury, cognition, anxiety, depression, inflammation

Traumatic brain injury (TBI) is a critical public health and socio-economic problem worldwide. It is one of the leading causes of death and disability among children and young adults, and its incidence in the elderly population has been rising (1). Data from the Centers for Disease Control and Prevention (CDC) indicate that each year in the USA 1.7 million people have a TBI, with 1.4 million of these injured individuals treated in emergency departments, leading to around 275,000 hospitalizations and 52,000 fatalities. The cost of TBI in the USA was estimated to be US$ 76.5 billion in 2010, comprising US$ 11.5 billion in direct medical expenses and US$ 64.8 billion with indirect expenses (2). High-quality data from low- and middle-income countries are scarce. In Brazil, it was recorded approximately 125,000 hospital admissions due to TBI per year between the years of 2008 and 2012, an incidence of 65.7 admissions per 100,000 inhabitants per year with a hospital mortality of 5.1/100,000/year (Information Technology Department of the Unified Health System, DATASUS). The average annual cost of these hospital expenses was US$ 70,960,000, with an average cost per admission of US$ 568. More importantly, a reliable quantification of the societal and personal burden caused by TBI in developing countries like Brazil is very difficult to establish due to inadequate standardization and incomplete capture of data on the incidence and outcome of brain injury (3).

Motor and cognitive deficits are well-known sequelae of TBI, contributing to long-term functional impairment and decrease in quality of life (4). Over the past years, studies have shown that behavioral or psychiatric disorders, specially depression and anxiety, also represent a significant source of distress in TBI patients (5). The prevalence of psychiatric diagnoses in these patients is much higher than in the general population, and up to 45% of them presents more than one psychiatric disorder. The detection and management of neuropsychiatric phenomena affects important decisions that will set the pace for treatment and increase the likelihood of successful rehabilitation (6). Accordingly, a careful assessment of neuropsychiatric disorders is essential in TBI.

Although molecular and cellular mechanisms involved in TBI pathophysiology are not fully understood, a growing body of evidence supports the involvement of a wide range of pathways including inflammatory response, oxidative stress, and excitotoxicity, that are associated with cognitive, motor, and behavioral outcomes (7–13). For instance, TBI induces a complex inflammatory response characterized by fast proliferation and migration of, respectively, brain resident immune cells (microglia) and circulating monocytes to the lesion site, with consequent production of inflammatory mediators that influence neuronal activity (7, 12). Microglial cells remain activated for weeks, months, or even years following a TBI event, which has been associated with increased expression of inflammatory cytokines, such as IL-1β and TNF, and long-term cognitive dysfunction (8–11). Therefore, TBI treatment should not focus only in the acute phase of the traumatic event but also on chronic processes that may influence the development of TBI long-term cognitive and behavioral changes (9, 14).

Currently, there is no treatment that prevents or minimizes TBI neurological and psychiatric sequelae. The understanding of the mechanisms underlying TBI pathophysiology may pave the way for the identification of biomarkers and potential therapeutic targets that ultimately will lead to efficacious therapeutic strategies (15).

This Research Topic gathers a set of seven studies in an interdisciplinary platform that addresses potential cellular and molecular mechanisms underlying TBI neuropsychiatry outcomes and potential therapeutic strategies. This Research Topic also highlights TBI-associated cognitive and behavioral changes in different populations as well as the continuing effort to identify predictors of TBI related mortality.

Areas et al. conducted a prospective multicentric study in Brazil to investigate predictors of hospital mortality in patients with severe TBI. During a period of 22 months, from April 2014 to January 2016, 266 patients with Glasgow coma scale (GCS) ≤ 8 were enrolled in the study. Patients were mostly men (*n* = 230, 86.5%) with a mean age of 38 years. The hospital mortality was 31.1% (*n* = 83). The study concluded that older age, worse neuroimaging results, as assessed by the Marshall Computed Tomography Classification, lower GCS scores, presence of subarachnoid hemorrhage in the CT, and pupillary abnormalities were independent predictors of hospital mortality.

Cardoso et al. investigated cognitive impairment in patients with mild TBI (GCS score ranging from 13 to 15), admitted at a different trauma reference in Brazil during the acute phase (<24 h) of the trauma. Compared to controls and normative data, TBI patients performed worse in a battery of cognitive tests, revealing significant cognitive deficits as memory and learning impairment. Interestingly, the cognitive alterations were not associated with loss of consciousness or previous TBI history, but seem to be negatively influenced by lower educational levels. This is one of the first studies to highlight the role of “cognitive reserve” in TBI outcomes.

Sports are important cause of TBI, and related concussions have been associated with short- and long-term cognitive impairment (16). Rodrigues et al. investigated whether subconcussive impacts occurring during soccer headings may lead to cognitive deficits in active professional soccer players. This type of impact may represent an additional mechanism of cumulative brain injury. Compared with non-athletes, matched by age, and educational level, no evidence of cognitive dysfunction was found in active professional soccer players. Future studies, with a longitudinal design and neuroimaging assessment will help to elucidate whether soccer heading might be a cause of brain injury and cognitive impairment.

Tau pathology seems to be one of the hallmarks of chronic traumatic encephalopathy. Katsumoto et al. revised the emerging evidence regarding clinical outcomes and molecular mechanisms underlying tau pathology following single and repetitive TBI. The authors also discussed the similarities and differences regarding the tau pathology observed in chronic traumatic encephalopathy and Alzheimer's disease. The mechanism of rapid tau accumulation remains to be unveiled in both conditions, but structural diversity of tau might explain, at least in part, the difference in outcomes.

Tapp et al. provided an overview regarding the role of neuroinflammation, mainly focused in microglia, in TBI acute and chronic neuropsychiatric sequelae. The authors highlight the involvement of physical and psychological stressors in triggering TBI cognitive and behavioral outcomes. They also hypothesize that the hypothalamic-pituitary-adrenal axis dysfunction following TBI might be a key factor underlying the emergence of neuropsychiatric symptoms through the activation of stress-immune pathways.

Besides cognitive impairment, mild TBI seems to be associated with dizziness and balance impairment. Lee et al. provided the first evidence that genetic variants of Acid-sensing ion channel 3 (ASIC3), an ion channel that responds to changes in pH and mechanical stimulation and is expressed in the vestibular and proprioceptive systems, may underlie mild TBI-associated balancing disorders. This finding may prompt further investigation of ASIC3 as a target for balance disorders therapeutics.

A single experimental study was conducted. Szczygielski et al. by employing a closed head injury model of severe TBI in mice demonstrated that administration of acetazolamide (ACZ), a carbonic anhydrase inhibitor, as an adjuvant strategy to decompressive craniectomy, attenuated brain edema, and neurological dysfunction. Further studies are necessary to explore the mechanisms underlying ACZ beneficial effects when combined with decompression following TBI, and to translate this finding into clinical practice.

We hope that this Research Topic might contribute to advance the understanding of such a complex and multifaceted field. We are very grateful to the different groups who submitted their scientific findings to this issue, and to the reviewers who kindly provided their time, effort, and experience to improve the quality of each study.

## Author Contributions

AM: conception, drafting the work, revising it, and final approval of the version to be published. LS and AT: conception, revising the work critically for important intellectual content, and final approval of the version to be published.

### Conflict of Interest

The authors declare that the research was conducted in the absence of any commercial or financial relationships that could be construed as a potential conflict of interest.
